# *Lavandula angustifolia* as a dual pharmacological system: from essential oil to polyphenol-rich biomass

**DOI:** 10.1080/13880209.2026.2679806

**Published:** 2026-06-05

**Authors:** Viktoriia Nechepurenko, Victoriya Georgiyants, Yuliya Prokopenko, Olha Mykhailenko

**Affiliations:** Department of Pharmaceutical Chemistry, National University of Pharmacy, Kharkiv, Ukraine

**Keywords:** *Lavandula angustifolia*, polyphenols, essential oil, pharmacology, valorization

## Abstract

**Context:**

*Lavandula angustifolia* Mill. is a widely used medicinal and aromatic plant with well-documented pharmacological activity, primarily attributed to its essential oil. However, the contribution of nonvolatile constituents and post-distillation biomass remains insufficiently characterized despite growing evidence of their biological relevance.

**Objective:**

This study aimed to provide a structured narrative synthesis of the phytochemical composition, pharmacological properties, and translational relevance of *L. angustifolia*, with particular emphasis on polyphenol-rich aerial biomass as an underexplored bioresource.

**Methods:**

A structured narrative review was conducted using ScienceDirect, Google Scholar, Scopus, PubMed, and SpringerLink databases through an iterative literature retrieval and refinement process. Priority was given to recent peer-reviewed English-language journal articles, particularly those published after 2015, with selective inclusion of earlier foundational studies where relevant. Eligible sources included studies reporting phytochemical characterization, mechanistic, experimental, translational, or clinical pharmacological data, as well as investigations addressing post-harvest or post-distillation biomass utilization. Literature was screened and synthesized using a thematic analytical approach.

**Results:**

*L. angustifolia* demonstrates a dual pharmacological profile driven by both volatile and nonvolatile fractions. Essential oil is consistently associated with antimicrobial and anxiolytic effects, including evidence from clinical and mechanistic studies. In contrast, polyphenol-rich fractions exhibit pronounced antioxidant and anti-inflammatory activity through modulation of key signaling pathways. Post-distillation biomass was identified as a relevant and underutilized source of bioactive phenolics with potential for pharmaceutical and nutraceutical applications.

**Conclusion:**

The findings establish *L. angustifolia* as a pharmacologically relevant dual-use plant system integrating essential oil production with biomass valorization. This approach supports circular bioeconomy strategies and highlights the translational potential of nonvolatile fractions in the development of value-added therapeutic products.

## Graphical Abstract

**Figure:**
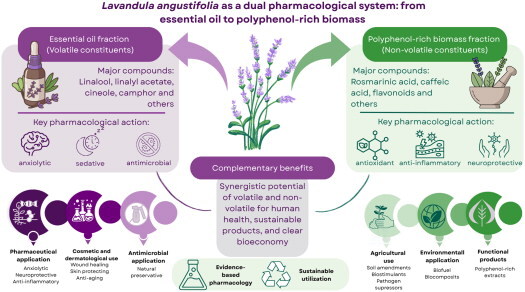

## Introduction

Lavender (genus *Lavandula* L., family Lamiaceae) is one of the most extensively studied aromatic and medicinal plants. Among its species, *Lavandula angustifolia* Mill. is particularly valued for the high quality of its essential oil and its long-standing use in traditional medicine, perfumery, and cosmetics. The genus originates from the Mediterranean region and comprises several dozen species and numerous hybrids, the most notable being *Lavandula × intermedia*, obtained through hybridization between *L. angustifolia* Mill. and *Lavandula latifolia* Medik.

In recent decades, *L. angustifolia* has transitioned from a primarily traditional and aromatic remedy to a clinically evaluated medicinal plant. This shift has been driven by increasing clinical and preclinical evidence supporting anxiolytic effects of standardized *L. angustifolia* essential oil preparations. Owing to its ecological plasticity and economic value, lavender is currently cultivated across a wide range of temperate regions worldwide. The leading producers of lavender essential oil remain Bulgaria and France; however, cultivation has expanded to North America, Africa, Australia, and various European countries, including Ukraine (Pokajewicz et al. 2022; Mykhailenko et al. 2025).

In Ukraine, lavender has not historically been considered a major medicinal crop. Nevertheless, interest in its cultivation has increased significantly in recent years. Traditionally, industrial plantations of essential oil crops, including lavender species, were concentrated in the southern regions, particularly in the Crimean Peninsula, as well as in the Mykolaiv and Kherson regions (Dementieva and Boiko 2021). Following the annexation of Crimea in 2014 and subsequent military events, a substantial proportion of these plantations was lost, creating the need to relocate and expand cultivation to other regions (Markovska et al. 2020).

Recent studies demonstrate that lavender can be successfully cultivated in multiple regions of Ukraine, including the southern steppe zone as well as selected western and central areas. New plantations have been established in regions such as Transcarpathia, Odesa, Kyiv, and Kharkiv, although their scale remains relatively limited (Kachanova et al. 2023; Kormosh et al. 2023; Bogatyrova et al. 2024; Svydenko et al. 2024). Despite ongoing geopolitical challenges, the expansion of lavender cultivation represents a promising direction for agricultural diversification and economic development.

At the same time, current production systems remain largely focused on essential oil as the primary product, while substantial amounts of post-harvest aerial biomass are generated and remain underutilized. This imbalance highlights a critical gap between agricultural production and downstream resource utilization. In this context, reconsidering *L. angustifolia* not only as a source of essential oil but also as a multifunctional plant resource, capable of providing both volatile and nonvolatile bioactive compounds, becomes increasingly relevant for both pharmacological research and sustainable bioeconomy development.

The aim of this study was to provide a structured narrative synthesis of the phytochemical composition, pharmacological properties, and translational relevance of *L. angustifolia*, with particular emphasis on the underexplored potential of post-harvest aerial biomass. The review seeks to integrate evidence on both volatile and nonvolatile fractions and to highlight opportunities for the valorization of lavender biomass within a sustainable, multi-sectoral framework.

## Materials and methods

This article was prepared as a structured narrative review focused on the phytochemical profile, pharmacological relevance, translational evidence, and post-harvest biomass valorization of *L. angustifolia*. The review followed an iterative structured narrative approach and did not employ a formal systematic review protocol, consistent with current methodological principles for rigorous narrative knowledge synthesis (Sukhera 2022).

Literature retrieval was conducted using ScienceDirect, Google Scholar, Scopus, PubMed, and SpringerLink databases. Priority was given to recent peer-reviewed English-language journal articles, particularly those published after 2015, that addressed the phytochemical composition, pharmacological properties, clinical relevance, or biomass valorization of *L. angustifolia*. Studies involving poorly defined “lavender oil” preparations without taxonomic or compositional clarification were not prioritized. Earlier studies were included selectively where necessary to provide foundational clinical, mechanistic, or phytochemical context, including seminal randomized controlled trials, foundational mechanistic investigations of anxiolytic activity, and key reports relevant to phytochemical characterization and biomass utilization.

Literature searches were last updated on 25 March 2026 prior to manuscript submission. Exact database-specific search strategies are provided in Supplementary Table S1.

Additional targeted iterative searches were performed during manuscript development to identify relevant literature for specific mechanistic, clinical, phytochemical, and biomass-related subtopics not fully captured by the core structured search strategy. Backward citation tracking of key review and primary research articles was additionally employed to identify relevant studies not captured through the initial database search.

A total of 414 preliminary records were identified through structured database searching and iterative targeted literature retrieval. Following consolidation and removal of duplicate records, 341 unique publications remained for screening. Literature selection and refinement were subsequently performed iteratively based on thematic relevance, methodological suitability, chemical specificity, and alignment with the review scope. The literature identification and refinement workflow is summarized in Supplementary Figure S1.

Eligible sources included peer-reviewed original articles and selected high-relevance reviews reporting at least one of the following: phytochemical characterization of *L. angustifolia* or its preparations, mechanistic, experimental, or translational pharmacological data, clinical evidence, or studies addressing utilization of post-harvest or post-distillation lavender biomass.

Preference was given to studies employing clearly defined botanical material, taxonomically identified as *L. angustifolia* Mill., and/or reporting sufficient compositional characterization of extracts or essential oils to support species attribution and interpretation of pharmacological relevance. Studies involving related *Lavandula* taxa or other Lamiaceae species were included only when direct *L. angustifolia*-specific evidence was limited and when such studies provided clearly identified comparative or mechanistic context; these are explicitly distinguished throughout the manuscript.

Sources were excluded if they lacked adequate phytochemical or compositional characterization of the investigated material, did not clearly specify botanical identity, were non-peer-reviewed, consisted of conference abstracts without sufficient methodological detail, duplicated previously reported findings without additional analytical value, or fell outside the pharmacological and biomass-valorization scope of the review. Secondary review articles were used selectively for contextual framing but were generally not prioritized as primary evidence sources when original literature was available.

The final literature corpus comprised 131 references selected for thematic synthesis and critical analysis. One additional methodological reference related to narrative review methodology was included in the final bibliography but was not counted as part of the thematic evidence synthesis (Sukhera 2022).

Graphical elements and schematic figures were prepared using the Canva online design platform. Botanical nomenclature and taxonomic validity were verified using the World Flora Online database (accessed March 2026).

## Results and discussion

### Phytochemical profile of Lavandula angustifolia

Medicinal and pharmacological relevance of *L. angustifolia* is determined by its chemically complex and heterogeneous phytochemical profile (Dobros et al. 2022b; Rathore and Kumar 2022). Although lavender essential oil has traditionally been considered the main source of biological activity, increasing evidence suggests that nonvolatile constituents may contribute to the broader biological activity profile of lavender-derived preparations, particularly in relation to antioxidant, antimicrobial, anti-inflammatory, and neuroprotective effects reported for polyphenol-rich extracts and related phytochemical fractions (López et al. 2017; Stanciu et al. 2019; Mykhailenko et al. 2025; Quoc et al. 2025). Phytochemical composition of *L. angustifolia* should therefore be regarded as a dynamic metabolic system influenced by ecological conditions, plant genotype, and extraction technologies. This complexity must be carefully considered when interpreting experimental pharmacological data, clinical outcomes, and the discrepancies reported across studies.

### Major groups of bioactive compounds

Bioactive constituents of *L. angustifolia* can be broadly divided into volatile compounds, primarily represented by essential oil components, and nonvolatile secondary metabolites, including polyphenols, coumarins, triterpenes, and tannins (Radu (Lupoae) et al. 2020). These groups differ in their chemical properties, pharmacokinetic behavior, and biological targets, while interactions between volatile and nonvolatile fractions may contribute to the overall therapeutic efficacy of lavender preparations (Bălașoiu et al. 2024; Sevindik et al. 2025).

#### Essential oil constituents

Volatile fraction of *L. angustifolia* is predominantly composed of monoterpenes and oxygenated monoterpenoids, which represent the major portion of lavender essential oil (Pokajewicz et al. 2022). Data from experimental GC-MS studies consistently confirm that monoterpenes constitute the dominant chemical class, with oxygenated monoterpenoids forming the major fraction (Dong et al. 2020). Along with linalool and linalyl acetate, other monoterpenes such as limonene, terpinen-4-ol, α-pinene, and β-pinene may also be detected as minor constituents, although typically at substantially lower concentrations than the dominant linalool/linalyl acetate chemotype components (Bogdan et al. 2020; Dong et al. 2020; Cheraif et al. 2022).

Literature sources have shown that qualitative and quantitative composition of lavender essential oil may vary considerably depending on geographical origin, environmental conditions, cultivation practices, plant genotype, and extraction methods (Hassiotis et al. 2014; Hassiotis and Vlachonasios 2025). For example, lavender cultivated in Mediterranean regions under traditional agronomic conditions is generally characterized by higher relative proportions of linalool and linalyl acetate, whereas cultivation under drought stress, elevated altitude, or non-native environmental conditions may shift terpene balance and increase the relative abundance of compounds such as terpinen-4-ol, camphor, or 1,8-cineole (Hassiotis et al. 2014; Bogdan et al. 2020; Hassiotis and Vlachonasios 2025). Environmental factors, therefore, influence not only total essential oil yield, but also the relative distribution of major and minor constituents within the volatile profile.

Chemotypic differentiation in *L. angustifolia* is primarily defined by the relative dominance of linalool and linalyl acetate, which represent the characteristic and most widely reported chemical profiles (Belhadj Mostefa et al. 2014; Stamova et al. 2025). These compounds are also widely recognized as the principal components and key quality markers of true lavender oil (Białoń et al. 2019; Pokajewicz et al. 2022; Jalil and Heinrich 2025). Nevertheless, some reports describe elevated levels of camphor or 1,8-cineole in samples identified as *L. angustifolia*, although such profiles should be interpreted cautiously, as they may reflect atypical chemotypes, environmental influences, harvesting-related variation, taxonomic ambiguity, or possible admixture/adulteration with related lavender taxa or lavandin-type material. Elevated camphor levels in particular are widely considered potential indicators of admixture with lavandin or spike lavender essential oils (Lafhal et al. 2016; Bejar 2020; Wilson et al. 2021).

However, phytochemical potential of lavender is not limited to its volatile fraction. A considerable proportion of biologically active metabolites is localized in the aerial parts of the plant and remains in the plant material after essential oil distillation. Consequently, lavender herb and post-distillation biomass have recently attracted attention as promising sources of nonvolatile bioactive compounds.

#### Polyphenolic compounds in lavender herb (aerial part)

Aerial parts of *L. angustifolia* contain a diverse range of polyphenolic constituents, including phenolic acids, flavonoids, and coumarins (Bogatyrova et al. 2024; Slavov et al., 2018). These compounds represent one of the most important groups of nonvolatile secondary metabolites and are considered likely contributors to the biological activity of polyphenol-containing lavender-derived preparations. Their occurrence and relative abundance may vary depending on plant organ, cultivar, environmental conditions, and extraction methodology, while leaves, flower buds, and flowers generally exhibit comparable levels of phenolic metabolites. In addition, the accumulation of these compounds may depend on the year of vegetation (Popoviciu and Panaitescu 2021).

Within the context of commercial lavender production, the relevance of these nonvolatile constituents becomes particularly evident. Flowers are primarily harvested for essential oil distillation, whereas a substantial amount of plant biomass remains as a by-product (Hassiotis et al. 2014). Considering that the essential oil content typically does not exceed about 1% of fresh biomass, large quantities of residual material remain after harvesting and distillation (Slavov et al. 2018). This biomass, referred to as lavender herb and consisting mainly of aerial parts, has traditionally been discarded or used as compost, despite its significant phytochemical potential.

Accumulating evidence indicates that post-harvest and post-distillation lavender herb retains considerable amounts of nonvolatile compounds, particularly phenolic acids, flavonoids, and coumarins. Identified phenolic acids include p-coumaric, neochlorogenic, caffeic, and rosmarinic acids, while flavonoids are represented by quercetin derivatives and myricetin (Méndez-Tovar et al. 2015; Adaszyńska-Skwirzyńska and Dzięcioł 2017; Slavov et al. 2018). Among these, rosmarinic acid is consistently reported as a predominant constituent and is often considered a characteristic marker of the polyphenolic fraction of *L. angustifolia*. Its presence, together with caffeic acid derivatives and luteolin, has been confirmed in post-distillation residues using aqueous ethanol extraction (Chilev et al. 2022), as well as in other chromatographic studies (Wang et al. 2026).

More detailed phytochemical investigations have revealed a broad spectrum of phenolic compounds present in lavender herb. These include cinnamic acid derivatives (caffeic, chlorogenic, p-coumaric, and ferulic acids), benzoic acid derivatives (gallic and ellagic acids), flavonols (hyperoside, isoquercitrin, quercetin, and rutin), and flavanols such as catechin and epicatechin (Ivanov and Vrancheva 2018). Advanced analytical techniques (e.g. HPLC-DAD) further indicate that rosmarinic acid, caffeic acid, and ferulic acid derivatives, particularly ferulic acid glucosides, represent major constituents, alongside flavonoids such as morin and coumarin derivatives, including coumarin and herniarin (Radi et al. 2021). Minor components such as ellagic acid, isoquercitrin, vanillin, and chlorogenic acid are typically present at lower concentrations, whereas caffeic, p-coumaric, neochlorogenic acids, and ferulic acid derivatives collectively form a substantial part of the phenolic acid pool (Vasileva et al. 2018; Kim et al. 2025a).

Further insight into the structural diversity of these compounds has been provided by high-resolution mass spectrometry (UHPLC-Q-Orbitrap HRMS/MS), which revealed numerous glycosylated derivatives (Çelik et al. 2017). A substantial proportion of phenolic acids occurs as O-glycosides, as evidenced by characteristic MS/MS fragmentation patterns involving the neutral loss of hexose moieties (162 Da), yielding diagnostic aglycone ions corresponding to caffeic, ferulic, and coumaric acids (Wang et al. 2026). Flavonoids are mainly represented by luteolin derivatives and their glycosylated forms, together with phenylpropanoid derivatives such as hydroxycoumarin and other minor secondary metabolites (Vasileva et al. 2018; Wang et al. 2026).

The recovery of polyphenolic compounds is strongly influenced by extraction conditions, including solvent composition, temperature, extraction time, and the number of extraction steps. A strong positive correlation between total phenolic content and antioxidant activity has been reported (Nurzyńska-Wierdak and Zawiślak 2016), with rosmarinic acid and caffeic acid derivatives identified as major contributors to antioxidant potential in lavender extracts, while flavonoids and coumarins are broadly recognized in phytochemical literature as compounds with anti-inflammatory and cytoprotective potential that may contribute to the observed activity profile (Kim et al. 2025b). At the same time, the overall pharmacological activity of lavender extracts may arise not only from individual compounds but also from synergistic interactions among different phytochemical constituents (Kozuharova et al. 2023; Bălașoiu et al. 2024).

Taken together, these findings highlight that lavender herb and post-distillation biomass represent an underutilized but valuable source of structurally diverse phenolic compounds with significant biological potential. Accordingly, lavender processing residues may serve as a promising raw material for obtaining bioactive compounds with applications in the food, cosmetic, and perfumery industries (Vasileva et al. 2018; Vareltzis et al. 2024), while alternative valorization strategies, including fermentation and biotransformation processes, have also been explored (Solomakou et al. 2025).

Several studies have investigated the potential use of polyphenol-rich extracts derived from lavender herb, particularly in relation to their antioxidant activity (Méndez-Tovar et al. 2015; Slavov et al. 2018; Mykhailenko et al. 2025; Aćimović et al. 2026), as well as the isolation and characterization of individual phenolic compounds. However, their practical application may be limited by relatively low stability, as these compounds can undergo degradation or structural transformation during processing, storage, transportation, and gastrointestinal digestion (Conte et al. 2023). Consequently, current research increasingly focuses on improving their stability, bioavailability, and shelf life.

Given the structural diversity of phenolic compounds in lavender biomass, the efficiency of their recovery is closely linked to extraction conditions, highlighting the importance of optimized extraction strategies for post-harvest lavender herb.

#### Other secondary metabolites

In addition to terpenoids and polyphenols, *L. angustifolia*, as well as other species, may contain smaller quantities of terpene compounds, coumarins, and tannins, which further enrich its phytochemical spectrum (Saadatian et al. 2013; Dobros et al. 2022a; Slimani et al. 2022; Fernane et al. 2023). Triterpenes, coumarins, and tannins identified in lavender and related plant matrices are pharmacologically relevant compound classes broadly associated in phytochemical literature with anti-inflammatory, antimicrobial, vascular, and tissue-repair activities; however, direct compound-specific evidence for these effects in *L. angustifolia* remains comparatively limited (Dobros et al. 2022a; Slimani et al. 2022). These compounds underscore the multifaceted chemical nature of *L. angustifolia* and highlight the importance of considering the full phytochemical spectrum, rather than isolated constituents, when evaluating its pharmacological activity and therapeutic applications (Sweeney et al. 2025). This integrated perspective provides the foundation for subsequent analysis of chemotypic variability and composition-activity relationships described in the following sections.

### Modern extraction techniques for polyphenolic compounds from post-harvest lavender herb

Efficient recovery of polyphenolic compounds from *L. angustifolia* biomass and related lavender processing residues requires the application of suitable extraction techniques. Since most phenolic constituents are nonvolatile and therefore do not participate in the essential oil distillation process, their extraction from lavender biomass has become an important focus of recent research. Various conventional and modern extraction methods have been investigated to obtain phenolic-rich extracts from lavender residues.

In recent years, modern extraction techniques have gained increasing attention due to their improved efficiency and reduced processing time. Compared with conventional techniques such as maceration, these approaches generally require shorter extraction times, lower solvent consumption, and reduced energy input, while often providing improved extraction efficiency and reproducibility.

Among these methods, ultrasound-assisted extraction (UAE) has been widely investigated for the recovery of phenolic compounds from plant materials, including lavender biomass (Turrini et al. 2021). This technique is based on the propagation of ultrasonic waves through the extraction medium, which induces acoustic cavitation. The formation and collapse of microscopic bubbles generate mechanical forces that disrupt plant cell structures and facilitate the release of intracellular metabolites into the solvent. As a result, ultrasound treatment enhances solvent penetration into plant tissues and improves the extraction of phenolic compounds (Vinatoru et al. 2017; Gonçalves and Romano 2021; Shen et al. 2023). UAE has been shown to be an effective approach for obtaining polyphenol-rich extracts from lavender residues while significantly reducing extraction time and solvent consumption compared with conventional extraction methods. Several studies have demonstrated the effectiveness of UAE for the recovery of phenolic compounds from lavender biomass. For example, Ivanov and Vrancheva (2018) reported that ultrasound-assisted extraction significantly improved the recovery of phenolic acids and flavonoids from lavender distillation residues compared with conventional maceration methods. Similarly, other authors have shown that UAE combined with hydroethanolic solvents enables efficient extraction of major phenolic constituents, including rosmarinic acid and caffeic acid derivatives (Ivanov and Vrancheva 2018).

Microwave-assisted extraction (MAE) represents another promising technique for the isolation of phenolic compounds from plant materials (Vinatoru et al. 2017). Microwave radiation rapidly heats both the solvent and plant matrix, accelerating the diffusion of target compounds into the extraction medium. Due to their higher dielectric properties, polar solvents are most frequently applied in MAE, which enables efficient absorption of microwave energy and rapid heating of the extraction system (Pavlić et al. 2019; Vareltzis et al. 2024). Experimental studies indicate that MAE enables rapid extraction of polyphenolic compounds from lavender residues while significantly reducing extraction time and solvent consumption, making it a promising alternative to conventional extraction methods (Vareltzis et al. 2024). As a result, MAE can significantly shorten extraction time and improve extraction efficiency. However, extraction parameters such as temperature and exposure time must be carefully controlled to avoid the thermal degradation of sensitive phenolic compounds.

Another advanced technique applied for the extraction of bioactive compounds from plant materials is supercritical fluid extraction (SFE). In this method, supercritical fluids exhibit physicochemical properties intermediate between those of liquids and gases, which allows efficient mass transfer and high extraction rates (Cruz-Sánchez et al. 2024). Carbon dioxide is the most commonly used supercritical solvent due to its nontoxicity, non-flammability, and relatively low critical temperature and pressure. Although supercritical CO_2_ is primarily suitable for the extraction of non-polar compounds such as lipids and essential oil components, the addition of small amounts of polar co-solvents, such as ethanol or water, allows the extraction of more polar metabolites, including polyphenolic compounds (Tyśkiewicz et al. 2019).

Beyond extraction itself, recent research has also explored strategies aimed at improving the stability and practical applicability of phenolic-rich extracts obtained from lavender by-products. Considering that different factors may affect polyphenolic compounds stability and biological activity, encapsulation technologies have been increasingly investigated as an approach to protect phenolic compounds and improve their functional properties (Zou et al. 2021; Solomakou et al. 2025).

Encapsulation techniques enable the stabilization and controlled release of bioactive compounds, thereby enhancing their bioavailability and extending their shelf life. Various approaches have been proposed for the stabilization of phenolic-rich extracts obtained from lavender residues, including spray-drying, freeze-drying, co-crystallization, and ionic gelation (Caser et al. 2023; Solomakou et al. 2025). These technologies allow the incorporation of bioactive compounds into protective matrices, improving their stability and facilitating their application as functional ingredients in food, pharmaceutical, and cosmetic products (Mehta et al. 2022). Overall, the combination of efficient extraction techniques with modern stabilization strategies represents a promising approach for the valorization of lavender biomass and the recovery of valuable phenolic compounds from lavender processing residues.

### Factors influencing phytochemical composition and strategies to enhance bioactive compound accumulation in lavender

Phytochemical composition of *L. angustifolia* exhibits considerable qualitative and quantitative variability, which may affect the reproducibility and comparability of pharmacological and clinical findings (López et al. 2017; Radu (Lupoae) et al. 2020). This variability arises from the combined influence of genetic, environmental, and technological factors, each of which modulates the biosynthesis, accumulation, and stability of both volatile and nonvolatile metabolites, including the polyphenolic compounds discussed above (Hassiotis et al. 2014; Ivanov and Vrancheva 2018; Kozuharova et al. 2023). As a consequence, lavender-derived materials of identical botanical origin may differ substantially in their chemical composition and, consequently, in their expected biological activity profiles.

At the structural level, plant organ represents a primary source of phytochemical variation, as they determine both the type and abundance of metabolites available for extraction. Flowering tops are typically enriched in essential oil constituents and phenolic compounds, whereas leaves and stems generally contain lower concentrations and altered ratios of key metabolites (Fakhriddinova et al. 2020; Wilson et al. 2021; Shaikh et al. 2026). Organ-specific differences have also been observed at the level of individual volatile constituents. For example, calyx tissues and flowering tops are typically enriched in linalool, linalyl acetate, lavandulyl acetate, and terpinen-4-ol, whereas leaves contain higher relative proportions of sesquiterpenes and compounds such as γ-cadinene, caryophyllene oxide, borneol, camphor, and 1,8-cineole, contributing to distinct aromatic profiles across plant tissues (Wilson et al. 2021). Similarly, phenological stage directly affects metabolite accumulation, as shifts in developmental priorities during flowering regulate carbon allocation toward terpene and phenylpropanoid pathways, thereby influencing both essential oil composition and phenolic content (Détár et al. 2021; Barut et al. 2022).

Environmental and climatic factors act as upstream regulators of secondary metabolism by modulating physiological stress responses and enzymatic activity within biosynthetic pathways (Hassiotis and Vlachonasios 2025). Temperature, solar radiation, altitude, soil composition, and water availability influence carbon partitioning, redox balance, and the expression of key enzymes involved in terpene and phenylpropanoid biosynthesis (Hassiotis et al. 2014; Ciesielska et al. 2020; Wilson et al. 2021; Saunier et al. 2022). As a result, environmental variation leads not only to changes in total metabolite yield but also to alterations in the proportions of individual constituents, thereby modifying the overall chemical profile (Coritar et al. 2019; Sağlam and Gidik 2025). Interannual climatic variability may further contribute to compositional fluctuations, as differences in temperature and precipitation between cultivation years have been associated with shifts in linalool, linalyl acetate, and terpinen-4-ol accumulation in *L. angustifolia* essential oil (Bogdan et al. 2020; Détár et al. 2020).

Among these factors, water availability represents a critical driver of metabolic reprogramming. Water deficit induces oxidative stress, which in turn activates defense-related pathways, including phenolic biosynthesis, which may promote increased accumulation of phenolic acids and flavonoids involved in antioxidant protection (Hosseini and Heidari 2025). At the same time, drought conditions modify terpene metabolism, resulting in changes in essential oil yield and in the balance between monoterpenes and oxygenated monoterpenes (Gorgini Shabankareh et al. 2026). Thus, water stress simultaneously reshapes both volatile and nonvolatile fractions of lavender phytochemistry.

Light conditions further influence metabolite accumulation through photoprotective and reactive oxygen species-mediated mechanisms (Ilić et al. 2026). Although direct evidence for *L. angustifolia* remains limited, studies on related Lamiaceae species, such as *Salvia rosmarinus* Spenn., demonstrate that increased light exposure, particularly UV radiation, enhances the accumulation of phenolic acids, including rosmarinic acid and caffeic acid derivatives, as part of adaptive antioxidant responses (Luis et al. 2007; Rahimi Rizi et al. 2023). Temperature exerts a dual effect by regulating enzymatic steps in terpene biosynthesis and influencing flower development, thereby indirectly controlling essential oil accumulation, while thermal stress can trigger compositional shifts in both volatile and phenolic fractions (Monaghan et al. 2004; Hassiotis et al. 2014; Hassiotis and Vlachonasios 2025).

Altitude and soil conditions further modulate phytochemical composition by integrating multiple environmental inputs. Altitude combines lower temperatures, increased UV exposure, and wider diurnal variation, thereby intensifying stress-related metabolic responses and leading to coordinated changes in both terpene and phenolic profiles (Demasi et al. 2018). Soil composition influences water retention, nutrient availability, and root-associated stress, which in turn regulate secondary metabolite pathways and contribute to variability in both essential oil composition and phenolic content (Chrysargyris et al. 2016; Najar et al. 2019; Zawadzińska et al. 2025).

In parallel, genetic background defines the intrinsic metabolic capacity of the plant, determining which biosynthetic pathways are preferentially activated. Chemotypic differentiation within *L. angustifolia* populations results in distinct chemical profiles, including variants characterized by high levels of esters such as linalyl and lavandulyl acetates, which are associated with high-quality essential oil (Despinasse et al. 2020).

Beyond intrinsic and environmental factors, technological processes directly affect the final chemical profile by modulating metabolite stability, recovery, and relative abundance. Cultivation practices, harvesting conditions, and post-harvest processing (e.g. drying and storage) influence degradation, transformation, or preservation of bioactive compounds, while extraction techniques and solvent selection determine the efficiency and selectivity of compound recovery (Hassiotis et al. 2014; Ilić et al. 2026). For example, hydrodistillation preferentially enriches volatile monoterpenes, whereas hydroethanolic and other polar extraction systems generally provide improved recovery of phenolic acids and flavonoids from lavender aerial biomass. In addition, microwave-assisted hydrodistillation has been reported to enhance cell disruption and mass transfer, resulting in differences in minor volatile constituents and increased recovery of phenolic compounds in post-distillation fractions compared with conventional hydrodistillation (Kırkıncı et al. 2024). These factors are particularly relevant for lavender herb valorization, as they shape the yield and composition of polyphenolic fractions highlighted in the previous section.

Given this multifactorial variability, recent research has shifted from descriptive analysis toward targeted modulation of phytochemical composition. Strategies aimed at enhancing and stabilizing bioactive compound accumulation focus on controlling the same factors that drive variability (El-Hefny and Hussien 2025; Spagnuolo et al. 2025).

Agronomic interventions, including optimization of irrigation regimes, fertilization strategies, planting density, and harvest timing, directly influence resource allocation and stress levels, thereby modulating secondary metabolism (Chrysargyris et al. 2016; Zawadzińska et al. 2025). Moderate abiotic stress and optimal harvesting during flowering have been shown to enhance both essential oil constituents and phenolic compounds, whereas excessive stress may reduce biomass and overall yield (Gorgini Shabankareh et al. 2026).

More advanced approaches, such as elicitation strategies and plasma-assisted treatments, aim to intentionally activate stress-response pathways and stimulate secondary metabolite biosynthesis (Georgiu et al. 2025; Hurina et al. 2025). In parallel, breeding and targeted selection enable long-term stabilization of desirable chemical profiles by selecting genotypes with consistent metabolite production (Zhelyazkova et al. 2025).

Importantly, enhancement of metabolite accumulation must be coupled with appropriate downstream processing, as increased biosynthesis translates into pharmacological relevance only if compounds are efficiently extracted and preserved (Georgiu et al. 2025; Spagnuolo et al. 2025).

Taken together, phytochemical profile of *L. angustifolia* reflects a dynamic interaction between environmental inputs, genetic predisposition, and technological interventions. While these factors introduce variability, they also provide opportunities for controlled modulation of both volatile and polyphenolic fractions, thereby enabling optimization of chemical composition with potential implications for the biological activity of lavender-derived preparations. This integrative perspective establishes a direct link between phytochemical variability and the pharmacological potential of lavender-derived preparations.

## Phytochemistry-pharmacology relationships

Pharmacological diversity of *L. angustifolia* cannot be adequately interpreted without consideration of its underlying phytochemical heterogeneity. Variations in the composition of nonvolatile phenolic compounds present in the aerial parts, together with differences in essential oil profile and environmentally driven shifts in metabolite accumulation, are considered important determinants of the biological activity profile of lavender-derived preparations. Rather than representing isolated effects of individual constituents, many pharmacological outcomes are thought to reflect compositional patterns and potential synergistic interactions among multiple chemical classes.

In this context, the pharmacological effects of *L. angustifolia* can be conceptually interpreted as involving two complementary yet mechanistically distinct phytochemical fractions: volatile essential oil constituents and nonvolatile polyphenolic compounds. These fractions differ not only in chemical properties but also in pharmacokinetic behavior and biological targets, giving rise to distinct pharmacodynamic profiles.

Volatile constituents of lavender essential oil, predominantly oxygenated monoterpenes such as linalool and linalyl acetate, are primarily associated with relatively rapid-onset pharmacological responses. Their high volatility facilitates fast absorption *via* inhalation or dermal routes, resulting in immediate neuropsychological and sensory effects (Linck et al. 2010; Lee et al. 2018; Oguro et al. 2025). Linalool has been extensively investigated for its neuropharmacological activity, including anxiolytic, sedative, and analgesic effects associated with modulation of central nervous system signaling, whereas linalyl acetate contributes to smooth muscle relaxation and calming properties (Koto et al. 2006; Linck et al. 2010; Caputo et al. 2018). These compounds have been linked to modulation of inhibitory neurotransmission, including GABAergic and serotonergic pathways, which is proposed to contribute to their anxiolytic and calming effects reported in experimental and clinical studies (Kazemi and Soureshjani 2014; López et al. 2017; Hashimoto et al. 2023).

A consistent trend can be observed in studies addressing compositional variation, where essential oil profiles enriched in linalool and linalyl acetate are more frequently associated with anxiolytic and sedative properties, whereas profiles containing higher proportions of camphor and 1,8-cineole have been linked to stronger antimicrobial and penetration-enhancing effects (Moghimi et al. 2015; Xie et al. 2016; Cardia et al. 2018; Harada et al. 2018; Hashimoto et al. 2023; Speranza et al. 2023; Quoc et al. 2025). However, interpretation of such profiles requires caution, as elevated camphor and cineole levels may also reflect taxonomic overlap, atypical chemotypes, or admixture with related Lavandula taxa or lavandin-type material (Lafhal et al. 2016; Bejar 2020; Pokajewicz et al. 2022). Thus, the biological activity of lavender essential oil is better interpreted in terms of compositional patterns rather than individual constituents. (Moghimi et al. 2015; Xie et al. 2016; Cardia et al. 2018; Harada et al. 2018; Hashimoto et al. 2023; Speranza et al. 2023; Quoc et al. 2025).

Molecular docking studies provide additional theoretical support for the potential ability of volatile terpenoids to directly interact with biological targets. *In silico* analyses have demonstrated that major essential oil components, including linalool, linalyl acetate, and β-caryophyllene, can bind to receptor sites such as NMDA subunits, suggesting potential modulatory or inhibitory effects. In addition, interactions with microbial enzymes, including DNA gyrase and elongation factor EF-Tu, have been reported, supporting the antimicrobial activity of lavender essential oil. Notably, even minor constituents may contribute to biological effects through favorable binding interactions, further emphasizing the importance of compositional complexity (Kazemi and Soureshjani 2014; Ousaid et al. 2024).

In contrast to the rapid and often transient effects associated with volatile constituents, nonvolatile polyphenolic compounds present in lavender aerial parts have been associated predominantly with sustained and cumulative biological responses. Due to their lower volatility and higher chemical stability, these compounds exhibit distinct pharmacokinetic behavior, enabling prolonged interaction with molecular targets and modulation of intracellular signaling pathways. As a result, polyphenolic compounds in lavender extracts have primarily been associated with long-term antioxidant, anti-inflammatory, and neuroprotective effects rather than immediate pharmacological responses (Stanciu et al. 2019; Slighoua et al. 2022; Hurina et al. 2025; Mykhailenko et al. 2025).

Biological activity of the polyphenolic fraction arises from the integrated action of multiple compounds rather than from a single dominant constituent. Phenolic acids such as rosmarinic, caffeic, and chlorogenic acids, together with flavonoids including luteolin derivatives, have been implicated in the modulation of oxidative stress, inflammatory signaling, and neuronal resilience. These effects are often cumulative and depend on repeated exposure, metabolic transformation, and interaction between compounds within the extract (Détár et al. 2020; Dobros et al. 2022b).

Molecular docking studies provide additional insight into the mechanisms underlying these effects. *In silico* analyses have demonstrated that key phenolic compounds, particularly rosmarinic acid and chlorogenic acid, have demonstrated predicted affinity toward acetylcholinesterase, suggesting a role in modulation of cholinergic neurotransmission. Although their interaction with NMDA receptor targets appears to be less pronounced compared to volatile constituents, compounds such as rosmarinic acid and luteolin still demonstrate measurable binding potential. These findings suggest that polyphenolic compounds exert their effects primarily through enzyme inhibition and regulation of intracellular pathways rather than direct receptor-mediated mechanisms (Mykhailenko et al. 2025; Cherbal et al. 2026).

Importantly, interactions between volatile and nonvolatile fractions may further influence the overall pharmacological profile of plant-derived preparations. Experimental studies on essential oil systems enriched with polyphenols have demonstrated that the addition of phenolic compounds or plant extracts can reduce antagonistic effects and enhance overall antioxidant activity, indicating the presence of synergistic interactions between different classes of phytochemicals (Nguyen and Karboune 2023). Such findings suggest that polyphenols may modulate the functional behavior of volatile constituents, thereby influencing the intensity and persistence of biological responses. These observations support the concept that the pharmacological activity of *L. angustifolia* arises from coordinated interactions between different classes of compounds rather than from isolated chemical entities.

**Figure 1.**
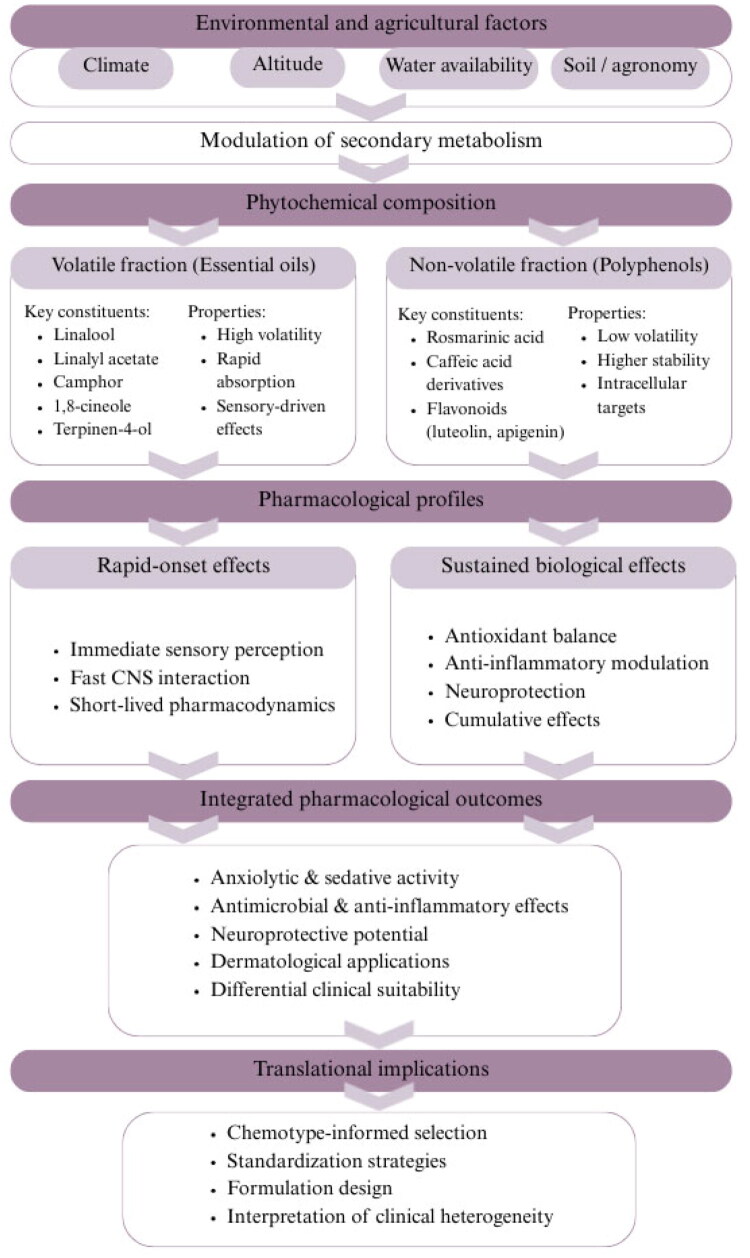
Integrated framework illustrating the contribution of volatile and nonvolatile fractions to pharmacological outcomes in *L. angustifolia.*

Taken together, these observations support the concept that lavender pharmacology reflects the coexistence of rapid, reversible effects mediated by volatile constituents and sustained biological modulation driven by polyphenolic compounds. This dual pharmacodynamic profile provides a mechanistic framework for interpreting variability across experimental and clinical studies and supports the rational design, standardization, and application of lavender-derived preparations. The scheme presented in Figure 1 provides an integrated conceptual framework linking environmental modulation, phytochemical composition, and pharmacological outcomes in *L. angustifolia*.

The scheme demonstrates that environmental and agronomic factors, including climate, altitude, water availability, and soil conditions, act as upstream regulators of secondary metabolism, shaping the overall phytochemical profile of the plant. This modulation is thought to contribute to the formation of two major phytochemical fractions with distinct physicochemical properties and biological roles: volatile essential oil constituents and nonvolatile polyphenolic compounds. These fractions differ in volatility, stability, and molecular targets, which likely contributes to differences in their pharmacokinetic behavior and pharmacodynamic effects.

Volatile constituents are characterized by rapid absorption and primarily contribute to immediate, short-lived pharmacological responses, particularly through interactions with the central nervous system and sensory pathways. In contrast, polyphenolic compounds exhibit lower volatility and higher stability, enabling prolonged interaction with intracellular targets and supporting sustained biological effects, including antioxidant, anti-inflammatory, and neuroprotective activities.

The coexistence of these two fractions gives rise to distinct yet complementary pharmacodynamic profiles, defined by rapid onset versus sustained biological responses. Their interaction is likely to influence the integrated pharmacological outcomes of lavender-derived preparations, including anxiolytic, antimicrobial, anti-inflammatory, and neuroprotective effects.

Importantly, this framework highlights that pharmacological activity should be interpreted as an emergent property of coordinated interactions between phytochemical composition, environmental modulation, and pharmacokinetic behavior. Such an integrated perspective provides a basis for chemotype-informed selection, standardization strategies, formulation design, and improved interpretation of variability across pharmacological and clinical studies.

Thus, *L. angustifolia* may be viewed as a useful model system in which pharmacological activity emerges from the dynamic interplay between rapid-acting volatile constituents and slowly acting nonvolatile compounds.

## Pharmacological evidence and translational relevance of *L. angustifolia*

### Mechanisms underlying anxiolytic activity

Experimental and translational studies indicate that the anxiolytic and mild sedative properties of *L. angustifolia* are thought to arise from coordinated modulation of multiple neurobiological systems rather than from interaction with a single molecular target (Tomi et al. 2018; Manzoor et al. 2025). This multimodal profile is consistent with the chemical complexity of lavender-derived preparations, although current mechanistic and clinical evidence is predominantly derived from essential oil-based preparations.

A substantial body of preclinical data implicates modulation of GABAergic neurotransmission as a central mechanism underlying lavender-induced anxiolytic and sedative effects, with reported effects including reduced sleep latency, increased NREM sleep duration, and enhanced EEG slow-wave following inhalation of lavender essential oil (25%) (Brum et al. 2001; Ren et al. 2025). Electrophysiological and pharmacological findings suggest facilitation of GABAA-mediated currents through non-benzodiazepine-associated mechanisms, potentially contributing to increased chloride conductance and neuronal hyperpolarization (Richardson et al. 2024). This mode of action is consistent with the observed *in silico* ability of lavender to modulate central nervous system activity *via* GABA_A_ receptors, which may underlie its reported sedative and neuroactive effects (Kazemi and Soureshjani 2014).

Behavioral studies employing validated anxiety paradigms, including the rotarod test, habituation/dishabituation test, and light-dark box test, consistently demonstrate anxiolytic-like effects following administration or inhalation of lavender essential oil or isolated linalool. In particular, exposure to linalool vapor for 30 min significantly increased time spent in the light compartment and the number of light-dark transitions (*p* = 0.0010 and *p* = 0.0029, respectively), with effects comparable to diazepam (1.5 mg/kg, i.p.), while no impairment of motor coordination was observed (*p* = 0.906) (Harada et al. 2018). These effects are attenuated by pharmacological antagonism of GABA_A_ receptors, supporting the involvement of inhibitory pathways in the observed behavioral responses (Kazemi and Soureshjani 2014). However, variability in experimental design, dosing, and exposure routes indicates that GABAergic modulation represents a major, but not exclusive, contributor to the overall anxiolytic profile.

In addition to inhibitory signaling, serotonergic pathways have been implicated in lavender-induced anxiolysis. Evidence for involvement of dopaminergic and noradrenergic systems is less consistent and appears to reflect secondary modulation rather than primary pharmacological targets (López et al. 2017).

Lavender-derived preparations have also been reported to influence stress-related neuroendocrine responses, including attenuation of hypothalamic-pituitary-adrenal (HPA) axis activation and reduction of stress-induced glucocorticoid levels. At the systems level, these effects are accompanied by decreased autonomic arousal, reflected in reduced heart rate and sympathetic tone. Together, these findings support a model of stress-response normalization rather than nonspecific central nervous system depression (Kasper et al. 2014; Dold et al. 2023).

At a network level, lavender appears to modulate limbic structures involved in emotional processing, including the amygdala and hippocampus. Importantly, these effects occur without marked impairment of cognitive or motor function, indicating selective targeting of dysregulated neural circuits associated with anxiety (Chambali and Algristian 2025).

Overall, preclinical evidence supports a model in which *L. angustifolia* preparations exert anxiolytic and mild sedative effects that are likely mediated through coordinated modulation. While these mechanisms provide a biologically plausible basis for clinical observations, their translational validation is currently confined primarily to standardized essential oil formulations, and the contribution of nonvolatile fractions remains insufficiently explored.

### Clinical evidence for anxiolytic effects

Clinical evidence supporting the anxiolytic and mild sedative effects of *L. angustifolia* remains predominantly derived from studies investigating standardized oral essential oil formulations, most notably Silexan. Randomized, double-blind clinical trials conducted across different patient populations have consistently reported statistically and clinically significant reductions in anxiety severity, including in subthreshold anxiety, generalized anxiety disorder, and mixed anxiety-depressive states. In a pooled analysis of five randomized, placebo-controlled trials (*n* = 1213), treatment with Silexan (80 mg per day for 10 weeks) resulted in a significant reduction in Hamilton Anxiety Rating Scale scores, with a responder rate ratio of 1.34 and a CGI-based improvement rate ratio of 1.51 compared to placebo, without an increased incidence of adverse events (Kasper et al. 2014; Dold et al. 2023).

Across these studies, fixed-dose administration of lavender oil (typically 80–160 mg per day) was associated with improvements in validated psychometric outcomes, including Hamilton Anxiety Rating Scale scores, as well as secondary parameters such as sleep quality and somatic symptoms. Importantly, these effects were achieved without clinically relevant sedation, cognitive impairment, or psychomotor slowing, supporting a comparatively favorable tolerability profile relative to conventional anxiolytic agents (Friedland et al. 2021; Volz and Klement 2022; Marchevsky 2024).

However, it is essential to emphasize that this body of evidence is largely restricted to essential oil-based preparations with defined chemical composition and dosing regimens. As such, the observed clinical efficacy reflects the pharmacological activity of specific volatile fractions rather than the whole phytochemical spectrum of *L. angustifolia* (López et al. 2017; Slimani et al. 2022). Direct extrapolation of these findings to crude extracts or preparations derived from lavender aerial parts is therefore not currently justified.

In contrast, clinical data specifically addressing nonvolatile fractions, including polyphenol-rich extracts of lavender herb, remain extremely limited. Despite preclinical evidence suggesting that polyphenolic compounds or lavender herb extracts may contribute to antioxidant, anti-inflammatory, and neuroprotective mechanisms, their clinical relevance in the context of anxiety-related disorders has not yet been systematically evaluated (Kaka et al. 2016; Ivanov and Vrancheva 2018; Dobros et al. 2022b; Slighoua et al. 2022). Available human evidence involving non-essential-oil lavender preparations remains fragmentary and insufficient for definitive conclusions, consisting primarily of small exploratory studies or formulations in which the contribution of polyphenolic fractions cannot be clearly separated from volatile constituents (Dobros et al. 2022a). This discrepancy highlights a significant gap between mechanistic understanding and clinical validation. Relevant preclinical evidence supporting antioxidant, anti-inflammatory, and neuroprotective mechanisms of lavender-derived polyphenolic fractions is discussed in greater detail in Section 4.3.

The above-mentioned studies employing inhalation-based or aromatherapy approaches represent an additional but highly heterogeneous body of evidence. These interventions typically target situational anxiety, procedural stress, or sleep disturbances and vary widely in essential oil composition, exposure duration, and outcome assessment (Harada et al. 2018; Zangbar et al. 2024; Wang et al. 2025). As a result, their findings are best interpreted as context-dependent and not directly comparable to standardized oral formulations (Linck et al. 2010; Harada et al. 2018; Satou et al. 2024; Oguro et al. 2025).

Therefore, the available clinical evidence supports the anxiolytic efficacy of standardized *L. angustifolia* essential oil preparations only under well-defined conditions. At the same time, the absence of controlled clinical studies on lavender herb extracts or polyphenolic fractions underscores the need to expand research beyond volatile constituents. Future investigations should focus on standardized, composition-defined extracts of aerial parts to clarify the clinical contribution of nonvolatile compounds and to bridge the gap between phytochemistry and therapeutic application.

Overall, the available data indicate that clinical evidence for lavender remains strongly formulation-dependent and currently does not extend to nonvolatile fractions of the plant.

### Polyphenolic fraction and sustained biological effects

Polyphenolic constituents of *L. angustifolia*, including rosmarinic acid, chlorogenic acid, caffeic acid derivatives, and flavonoids such as luteolin glycosides, are widely associated with antioxidant and anti-inflammatory activities in studies of lavender-derived extracts and broader phytochemical literature, while their neuroprotective potential remains primarily supported by preclinical evidence (Dobros et al. 2022a; Slighoua et al. 2022; Mykhailenko et al. 2025).

A central mechanism underlying these effects is thought to involve regulation of oxidative stress. Polyphenolic compounds have demonstrated antioxidant-related activities, including reactive oxygen species scavenging, inhibition of lipid peroxidation, and enhancement of endogenous antioxidant defenses, as reflected by high ferric reducing antioxidant power (FRAP) values of lavender extracts, reaching up to 179.6 mg AAE/g DW and total polyphenol content up to 152.4 mg GAE/g DW depending on cultivar (Détár et al. 2020). These actions are consistent with improved cellular redox balance observed in experimental models, with a direct correlation observed between antioxidant activity and polyphenolic content, where increases in total polyphenols (up to 25%) are accompanied by enhanced radical scavenging activity (up to 32%) (Hurina et al. 2025).

Moreover, scientific reports suggest that lavender-derived polyphenolic fractions may modulate inflammatory signaling pathways. This is further supported by *in vivo* studies, where anti-inflammatory effects of lavender-derived hydroethanolic extracts (300–600 mg per kg) and polyphenolic fractions (100–200 mg per kg) after oral administration significantly reduced carrageenan-induced edema, with inhibition reaching 35–38% and 26–43%, respectively, at 3 h, and up to 68–72% and 56–76% at 6 h, compared to 84% for indomethacin (10 mg per kg) (Slighoua et al. 2022; Bohatyrova and Naboka 2025). Experimental studies have reported dose-dependent suppression of NF-κB activation, reduction of nitric oxide and pro-inflammatory cytokines (TNF-α, IL-6, IL-1β), enhancement of antioxidant defense (e.g. SOD activity), and modulation of MAPK-related signaling cascades, with biologically relevant effects observed at concentrations of 25–100 μg per mL (Wang et al. 2026). Through these mechanisms, lavender polyphenols may contribute to neuroprotection and enhance the brain’s resistance to neurodegenerative processes. This anti-inflammatory component may be particularly relevant in the context of chronic stress, neurodegeneration, and systemic inflammatory states (Bohatyrova and Naboka 2025; Mykhailenko et al. 2025). The involvement of lavender polyphenols in neuroprotective and neuromodulatory processes has been discussed above in section 3. «Phytochemistry-pharmacology relationships». Considering that polyphenols act through multiple mechanisms, including radical scavenging, modulation of oxidative stress, and regulation of inflammatory processes, lavender herb may be regarded as a promising source of bioactive compounds with potential pharmacological applications (Yahfoufi et al. 2018).

From a translational perspective, polyphenol-rich extracts of lavender aerial parts remain comparatively less explored in clinical research. While the majority of clinical evidence focuses on essential oil-based preparations, the mechanistic profile of nonvolatile constituents suggests their potential relevance for chronic and preventive applications. The absence of standardized clinical studies in this area highlights a critical gap and underscores the need to expand research beyond volatile fractions toward composition-defined extracts of lavender herb.

Taken together, these observations support the concept that the pharmacological profile of *L. angustifolia* may extend beyond rapid neurosensory effects and involve sustained biological modulation associated with its polyphenolic fraction. Integration of these nonvolatile components into future research frameworks will be important for achieving a more comprehensive understanding of lavender pharmacology and for bridging the gap between phytochemistry and long-term therapeutic effects.

### Peripheral and local biological effects

Peripheral pharmacological activities of *L. angustifolia* encompass antimicrobial, anti-inflammatory, antioxidant, and dermatological effects, which are thought to arise from the combined action of volatile and nonvolatile constituents. While essential oil components have been extensively studied in this context, increasing evidence indicates that polyphenolic compounds may contribute substantially to sustained peripheral biological responses associated with lavender-derived preparations (Stanciu et al. 2019; Bălașoiu et al. 2024; Bogatyrova et al. 2024; Hosseini and Heidari 2025).

Antimicrobial activity has been widely reported *in vitro*, with essential oil constituents with proposed membrane-disruptive effects against a range of bacterial and fungal species. Behmanesh et al. (2015) report that lavender essential oil has shown inhibitory effects against *Candida albicans*, with reduced fungal cell counts observed at dilutions of 1/20 to 1/160 after 24 h of exposure (p < = 0.02), although these effects were not maintained after prolonged incubation (Behmanesh et al. 2015). In addition, other studies demonstrate that essential oil from *L. angustifolia* exhibits broad-spectrum antibacterial activity, with MIC values ranging from 0.31% to 10% depending on the strain, and pronounced bactericidal effects in time-kill assays, including > =3 log10 CFU/mL reduction within 8 h at 0.62% and near-complete bacterial eradication at 1.24% within 6 h (Stamova et al. 2025). It is also worth mentioning that polyphenolic compounds may contribute indirectly to antimicrobial activity by modulating oxidative balance and enhancing host defense mechanisms, although their direct antimicrobial potency is generally lower (Ivanov and Vrancheva 2018; Mykhailenko et al. 2024).

Dermatological and wound-healing effects are likely to involve the convergence of antimicrobial, anti-inflammatory, and antioxidant mechanisms. In this context, polyphenols are thought to contribute to the regulation of inflammatory responses and oxidative balance within the skin microenvironment, while essential oil constituents may contribute to local antimicrobial activity and penetration enhancement. The local mode of application may enhance translational plausibility by enabling higher effective concentrations at the site of action (Mori et al. 2016; Zyburtowicz et al. 2024; Kim et al. 2025b).

Overall, the peripheral pharmacological activities of *L. angustifolia* are best understood as the result of complementary interactions between volatile and nonvolatile fractions. However, sustained and potentially systemically relevant effects may be more closely associated with polyphenolic constituents, highlighting the importance of considering lavender aerial parts as a pharmacologically relevant matrix beyond essential oil-based applications.

To provide a structured overview of pharmacological evidence, key findings across different biological activities, experimental models, and active fractions are summarized in Table 1. Information on dosing and experimental conditions is provided within the corresponding sections.

**Table 1.** Pharmacological evidence of *L. angustifolia*: integration of preclinical and clinical data.

Activity	Model	Fraction/compound	Key findings	References
Anxiolytic	Clinical (RCT, meta-analysis)	Essential oil (Silexan)	Significant reduction of anxiety symptoms compared to placebo and standard therapy	Kasper et al. 2014; Dold et al. 2023
Anxiolytic	*In vivo*	Linalool	Reduced anxiety-like behavior in elevated plus maze and social interaction tests	Linck et al. 2010; Harada et al. 2018
Neuroprotective	*In vivo*	Essential oil/linalool	Improved cognitive performance and reduced behavioral impairment	Lee et al. 2018; Ren et al. 2025
Neuroprotective	*In vitro*	Polyphenolic fraction	Reduction of oxidative stress and modulation of neuronal signaling pathways	Mykhailenko et al. 2025
Anti-inflammatory	*In vitro/in vivo*	Essential oil	Decreased pro-inflammatory mediators and inhibition of inflammatory pathways	Cardia et al. 2018
Antioxidant	*In vitro*	Polyphenol-rich extracts	Strong radical-scavenging activity and inhibition of lipid peroxidation	Dobros et al. 2022b; Ivanov and Vrancheva 2018
Antioxidant	*In vitro*	Rosmarinic acid, flavonoids	Maintenance of redox homeostasis and cellular protection	Yahfoufi et al. 2018
Antimicrobial	*In vitro*	Essential oil	Inhibition of bacterial and fungal growth	Behmanesh et al. 2015; Stamova et al. 2025
Dermatological/wound healing	*In vivo*	Essential oil	Acceleration of wound contraction and tissue regeneration	Mori et al. 2016
Skin recovery/antioxidant	*In vitro*	Leaf/callus extract	Activation of Nrf2 signaling and improved redox balance	Kim et al. 2025b

As summarized in Table 1, pharmacological evidence across different domains remains uneven, with comparatively well-established clinical support for anxiolytic effects contrasted by predominantly preclinical evidence for most other reported activities.

### Translational perspective and research gaps

The translational trajectory of *L. angustifolia* varies substantially across pharmacological domains and reflects a pronounced hierarchy of evidence. Among the reported activities, anxiolytic effects represent the pharmacological domain currently supported by the most complete translational continuum, integrating mechanistic plausibility, standardized formulation, reproducible dosing, and validation in randomized controlled clinical trials (Kasper et al. 2014; Friedland et al. 2021; Dold et al. 2023; Marchevsky 2024). In this context, standardized *L. angustifolia* essential oil preparations represent a notable example in phytotherapy where experimentally defined neurobiological mechanisms correspond to clinically measurable outcomes.

Beyond anxiolysis, translational progress remains partial and uneven. Dermatological and topical applications appear to demonstrate comparatively higher translational plausibility, largely due to favorable pharmacokinetic conditions, as local administration enables effective concentrations at the site of action while minimizing systemic constraints (Mori et al. 2016; López et al. 2017; Zyburtowicz et al. 2024). However, available clinical data in these areas remain limited in scale and methodological consistency.

In contrast, pharmacological activities associated with anti-inflammatory, antioxidant, antimicrobial, and neuroprotective effects are supported predominantly by preclinical evidence. Notably, these domains appear to be more closely associated with nonvolatile constituents present in lavender aerial parts, including polyphenolic compounds, which exhibit pharmacologically plausible and mechanistically coherent activity profiles but remain comparatively less characterized in clinical settings (Stanciu et al. 2019; Dobros et al. 2022b; Mykhailenko et al. 2025). The lack of standardized extract compositions, as well as limited pharmacokinetic characterization or the absence of biomarker-driven clinical studies, currently restricts the translation of these findings into evidence-based therapeutic applications.

This discrepancy reflects the current imbalance between phytochemical knowledge and clinical validation. While volatile constituents have been extensively investigated in controlled clinical frameworks, nonvolatile fractions derived from lavender aerial parts, including post-harvest biomass remaining after essential oil production, have only recently begun to attract broader translational interest. Importantly, this gap also reflects the limited consideration of lavender herb as a secondary resource, despite its retained bioactive potential and relevance for sustained biological effects. As a result, extrapolation from *in vitro* and experimental models to systemic therapeutic claims requires further validation before broader therapeutic extrapolation.

Future progress will depend on coordinated efforts to standardize plant-derived extracts, characterize pharmacokinetic behavior of both volatile and nonvolatile constituents, and implement clinical trial designs aligned with the underlying mechanisms of action. Particular importance should be placed on distinguishing short-term symptomatic effects from long-term disease-modifying potential, especially in contexts such as neuroinflammation and oxidative stress-related conditions. In parallel, integration of post-harvest lavender biomass into research and development frameworks may enable expansion of application domains beyond essential oil–based products and support more sustainable and resource-efficient utilization of the herb.

Overall, standardized *L. angustifolia* essential oil formulations can be regarded as clinically validated anxiolytic preparations under defined pharmaceutical conditions, whereas the broader pharmacological potential of lavender, particularly that associated with nonvolatile constituents of post-harvest aerial biomass, represents a mechanistically promising and increasingly investigated area of lavender research that still requires broader translational and clinical validation.

## Future directions and research gaps

Despite substantial advances in the phytochemical and pharmacological characterization of *L. angustifolia*, current research remains largely centered on essential oil as the primary product. In contrast, post-harvest aerial biomass generated after flower collection and distillation remains insufficiently explored, despite evidence indicating retention of diverse bioactive and structural components. Future progress will therefore depend not only on addressing methodological limitations but also on redefining lavender herb as a multifunctional resource within integrated production systems.

### Environmental variability and chemotype standardization

Environmental variability represents a major challenge for reproducibility in *L. angustifolia* research. Climate conditions, soil characteristics, and cultivation practices significantly influence the accumulation of both volatile and nonvolatile constituents, affecting essential oil profiles as well as the composition of residual aerial biomass. This variability complicates cross-study comparability and limits the predictability of downstream applications (Hassiotis et al. 2014; Dementieva and Boiko 2021; Hassiotis and Vlachonasios 2025).

Future research should prioritize longitudinal and multi-regional studies integrating environmental metadata with comprehensive phytochemical profiling of both essential oil and post-harvest plant material. Development of consensus-based chemotype frameworks that encompass the full phytochemical spectrum, including nonvolatile fractions, would improve reproducibility and enable more efficient and targeted utilization of lavender-derived resources.

### Expansion of application domains beyond essential oil-based anxiolysis

Current clinical and pharmacological research is largely confined to standardized essential oil preparations, particularly in the context of anxiolytic applications. While this represents a well-established translational pathway, it reflects only a limited fraction of the broader biological and functional potential of *L. angustifolia* (Kasper et al. 2014; Dold et al. 2023; Marchevsky 2024).

Future studies should extend beyond volatile constituents and incorporate the broader utilization of lavender aerial parts, including extracts derived from post-harvest biomass. Priority directions include dermatological, anti-inflammatory, antioxidant, and functional product applications, where sustained biological effects are pharmacologically plausible and supported primarily by preclinical and mechanistic evidence, but remain insufficiently validated in translational and clinical settings. Progress in these areas will require harmonization of extraction methods, formulation strategies, and application-specific evaluation frameworks.

### Characterization and standardization of post-harvest lavender biomass

Post-harvest lavender herb represents a complex and undercharacterized matrix containing polyphenolic compounds, residual secondary metabolites, and structural components with potential functional relevance. However, its composition remains highly variable, and systematic approaches to its characterization and standardization are currently lacking (Ivanov and Vrancheva 2018; Slavov et al. 2018; Aćimović et al. 2026).

Future work should focus on the development of integrated analytical frameworks for post-distillation biomass, including the identification of key marker compounds, optimization of extraction strategies, and evaluation of functional properties relevant to different application domains. In particular, polyphenolic fractions require further investigation with respect to their pharmacokinetic behavior and contribution to sustained biological effects.

Establishing standardized processing and characterization protocols will be important for facilitating the transformation of residual plant material into a more reproducible and scalable resource suitable for pharmaceutical, cosmetic, and industrial applications.

### Integration into sustainable bioeconomy frameworks

Beyond pharmacological applications, *L. angustifolia* represents a valuable resource within plant-based bioeconomy systems. Its multifunctional use across pharmaceutical, cosmetic, agricultural, and industrial sectors offers opportunities for diversification of production and value chains (Aćimović et al. 2026).

A key direction involves chemotype-informed cultivation strategies, enabling alignment of specific phytochemical profiles with targeted applications. At the same time, cascading utilization of plant material offers substantial opportunities for improving resource efficiency. Post-distillation residues and aerial biomass have been reported to retain polyphenolic compounds and structural constituents with potential functional relevance that can be valorized through extraction, formulation, or incorporation into functional products, potentially contributing to waste reduction and improved resource efficiency (Pyrovolos and Kamperidou 2025).

Advances in green extraction technologies, controlled-release systems, and formulation approaches further support the transition from essential bulk oil production toward standardized, value-added products. However, such development requires consistent chemical characterization and integration of agronomic, technological, and pharmacological parameters to ensure both reproducibility and sustainability.

### Integrative perspective

Future development of *L. angustifolia* research can be understood as a transition from a single-product paradigm toward an integrated, multi-component utilization model. While anxiolytic effects of standardized essential oil preparations represent the current translational benchmark, substantial gaps remain in chemotype harmonization, environmental data integration, and clinical validation of nonvolatile fractions.

At the same time, increasing evidence suggests that post-harvest aerial biomass traditionally treated as a low-value by-product retains potentially valuable functional properties. Polyphenol-rich extracts derived from residual plant material have demonstrated antioxidant and anti-inflammatory activities in experimental studies, supporting their potential application in pharmaceutical, cosmetic, and nutraceutical contexts (Slavov et al. 2018; Stanciu et al. 2019; Mykhailenko et al. 2025). In parallel, lavender biomass may also be utilized in agricultural systems as soil amendments or biostimulants, and in industrial applications as a source of functional materials (Aćimović et al. 2026). Taken together, the valorization potential of post-harvest lavender biomass across different application domains is summarized in Table 2.

**Table 2.** Valorization pathways and application domains of post-harvest *L. angustifolia* biomass.

Application domain	Biomass/fraction	Key components	Functional properties/use	References
Antioxidant activity; potential cosmetic and pharmaceutical applications	Post-distillation biomass (ultrasound-assisted extraction, PUAE)	Polyphenols (gentisic acid, flavonoids incl. quercetin derivatives)	High recovery of antioxidant compounds; gentisic acid up to ∼10–13 mg/g DW; potential applications in cosmetic (skin-lightening) and pharmaceutical formulations	Turrini et al. 2021
Antioxidant and antimicrobial agents	Post-distillation residues (surfactant-assisted extraction)	Phenolic acids, flavonoids	Optimized extraction yield (52.2 mg GAE/g) with strong antioxidant activity (DPPH IC₅。 ≈ 0.45 mg/mL) and selective antimicrobial effects against Gram-positive bacteria	Barar and Bensebia 2025
Food/cosmetic additives	Post-distillation (SD-L) and post-CO₂ extraction (CO₂-L) lavender biomass	Pectic polysaccharides (galacturonic acid-rich)	Foam-forming ability (higher in SD-L); emulsifying properties; viscosity modification	Marovska et al. 2022b
Antioxidant and antimicrobial activity; potential food applications (biopreservative)	Post-distillation biomass obtained from steam distilled lavender (SD-L) and from subcritical CO₂ extraction of lavender (CO₂-L) (ethanolic extracts)	Phenolic acids (*p*-coumaric, caffeic, neochlorogenic, rosmarinic acids), flavonoids (catechin, quercetin derivatives)	Strong antioxidant activity (ORAC, HORAC, DPPH, FRAP assays); antimicrobial potential; higher phenolic content in SD-L; potential use as low-cost biopreservative in food systems	Slavov et al. 2018
Waste valorization/organic fertilizer (compost production)	Lavender waste from essential oil extraction (vermicomposting with *Eisenia andrei*)	Not specified (organic biomass)	Efficient conversion into compost; enhanced earthworm growth and reproduction; nontoxic (germination index >100); potential use as organic fertilizer without pretreatment	González-Moreno et al. 2022
Biofuel production (solid fuel pellets)	Post-distillation lavender biomass (blended with fir wood for pelletization)	Not specified (lignocellulosic biomass)	Suitable for pellet production with adequate calorific value (∼17.9 MJ/kg); meets quality standards (moisture, density, durability); potential alternative feedstock for solid biofuels	Pyrovolos and Kamperidou 2025
Functional materials (biochar-based materials)	Lavender agro-industrial residues (carbonized and Zn²⁺-modified biochar)	Carbon-based material (biochar) with Zn²⁺ incorporation	Structurally stable and homogeneous biochar with improved surface properties (porosity, stability, potential conductivity); suitable as a platform for further functional applications	Sandov et al. 2026
Waste valorization (composting)	Post-distillation lavender waste (co-composting with manure and straw; bacterial inoculation)	Not specified	Suitable for compost production; supports organic matter degradation and compost maturation; potential negative effects (e.g. increased C/N ratio) mitigated by microbial inoculation; production of stable, pathogen-free compost	Greff et al. 2021

The data presented in Table 2 illustrate the multifunctional nature of post-harvest lavender biomass and its applicability across diverse sectors. These findings support the concept of lavender herb as a valuable secondary resource rather than a low-value by-product. At the same time, the heterogeneity of reported applications highlights the need for harmonized processing and characterization approaches. These directions align with circular bioeconomy principles, where waste streams are redefined as valuable resources. However, their effective implementation requires coordinated optimization of processing technologies and integration of downstream applications into existing production chains.

**Figure 2.**
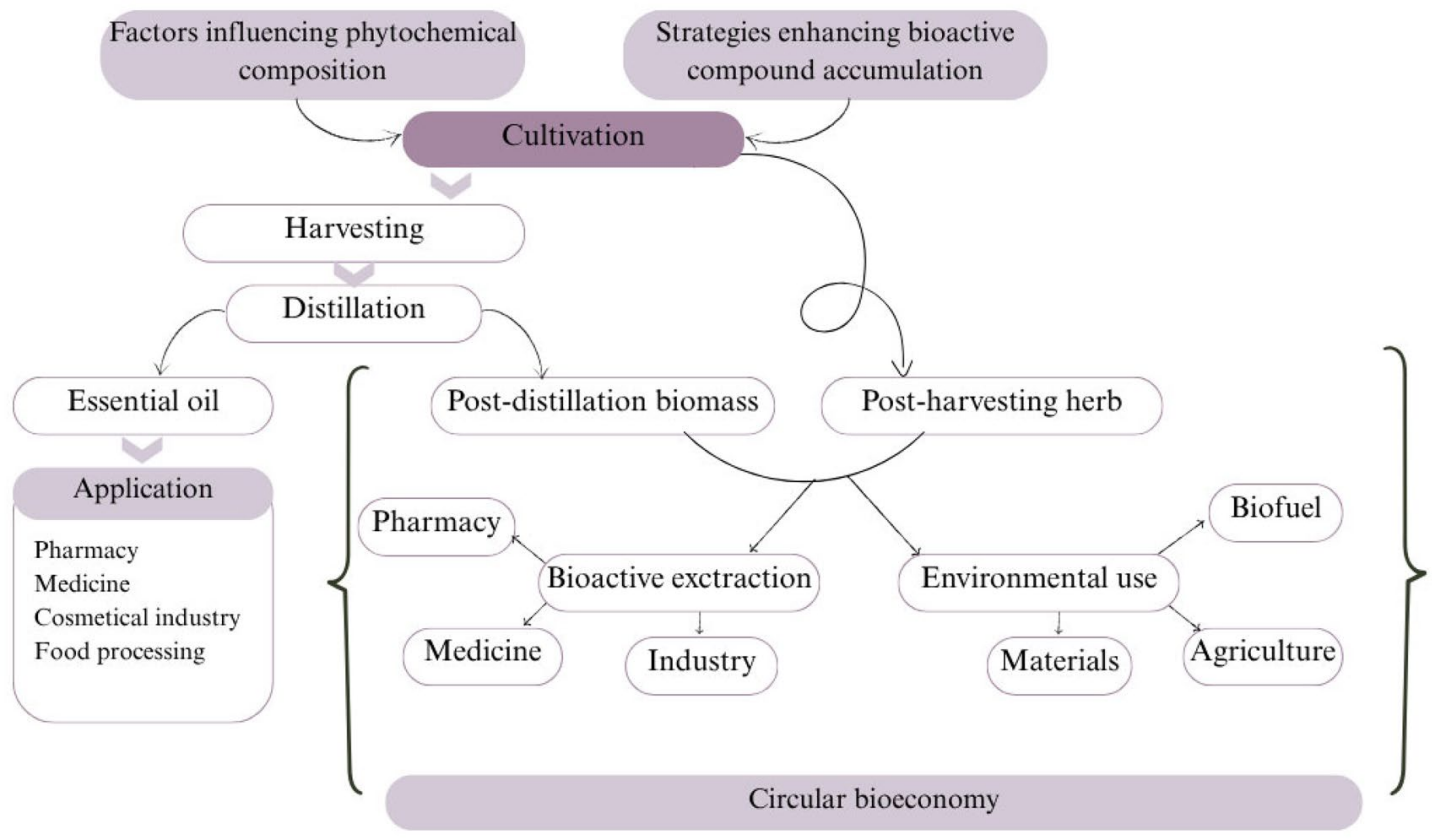
*L. angustifolia* as a dual phytochemical and bioresource system.

Overall, future progress is likely to depend on a shift from descriptive and compartmentalized research toward more integrated, mechanism-driven, and sustainability-oriented strategies, in which the valorization of post-harvest lavender biomass becomes central to redefining *L. angustifolia* as a multifunctional and resource-efficient bioresource (see Figure 2).

The proposed scheme conceptualizes *L. angustifolia* as a dual phytochemical and bioresource system, integrating essential oil production with the valorization of polyphenol-rich post-harvest biomass. It highlights the shift from linear processing toward circular bioeconomy strategies, where residual plant material is repurposed into value-added products. This framework is intended to support a more complete and sustainable utilization of lavender resources.

## Conclusions

Phytochemical profile of *L. angustifolia* may be regarded as a complex and dynamic system in which volatile and nonvolatile constituents are associated with distinct yet potentially complementary biological activities. While essential oil components are primarily associated with terpene-driven effects, the aerial plant parts and post-distillation biomass represent a rich source of structurally diverse polyphenolic compounds, including phenolic acids and flavonoids, which are widely associated with antioxidant, anti-inflammatory, and cytoprotective activities in experimental and phytochemical studies.

Modern extraction techniques, combined with controlled agronomic and technological conditions, may enable improved recovery and stabilization of polyphenolic compounds. At the same time, genotype-environment interactions and processing parameters must be carefully considered to ensure reproducibility, standardization, and more reproducible pharmacological profiles of lavender-derived preparations.

The nonvolatile fraction of *L. angustifolia* represents a potentially important source of biologically active compounds with pronounced antioxidant and anti-inflammatory properties. The recovery and utilization of polyphenol-rich residual biomass after essential oil distillation provide an effective strategy for extending the pharmacological value of lavender beyond its volatile constituents.

Pharmacological potential of *L. angustifolia* can be conceptually interpreted within a dual framework, where essential oil and polyphenol-rich biomass contribute complementary biological activities. While volatile compounds are primarily associated with neuroactive effects, the residual plant material constitutes a valuable source of polyphenols, supporting its application as a sustainable raw material in circular bioeconomy approaches.

Post-harvest aerial biomass of *L. angustifolia*, traditionally considered a low-value by-product, represents a multifunctional and underutilized source of bioactive and structural compounds. Its integration into value-added applications across pharmaceutical, cosmetic, agricultural, and industrial domains may support a transition toward circular bioeconomy-oriented utilization strategies.

## Supplementary Material

Supplemental Material

## Data Availability

All data supporting the results of this study are included in the manuscript, and the datasets are available upon request.
