# Supplementary material for: Lavandula angustifolia as a dual pharmacological system: from essential oil to polyphenol-rich biomass
Source: Pharm Biol. 2026 Jun 5;64(1):821–47. doi: 10.1080/13880209.2026.2679806 (PMC13244516; doi:10.1080/13880209.2026.2679806)
Supplement: Supplemental Material [file IPHB_A_2679806_SM1889.docx]

Supplementary Table S1

Database-specific literature search strategies used in the present structured narrative review

| Database | Search strategy | Last search date |
| --- | --- | --- |
| PubMed | (“Lavandula angustifolia”[Title/Abstract] OR lavender[Title/Abstract]) AND (“essential oil”[Title/Abstract] OR polyphenols[Title/Abstract] OR phenolics[Title/Abstract] OR flavonoids[Title/Abstract] OR biomass[Title/Abstract] OR “post-distillation residue”[Title/Abstract]) AND (pharmacology[Title/Abstract] OR anxiolytic[Title/Abstract] OR antioxidant[Title/Abstract] OR anti-inflammatory[Title/Abstract] OR antimicrobial[Title/Abstract] OR extraction[Title/Abstract] OR valorization[Title/Abstract]) | March 2026 |
| Scopus | TITLE-ABS-KEY (“Lavandula angustifolia” OR lavender) AND TITLE-ABS-KEY (“essential oil” OR polyphenols OR phenolics OR flavonoids OR biomass OR “post-distillation residue”) AND TITLE-ABS-KEY (pharmacology OR anxiolytic OR antioxidant OR anti-inflammatory OR antimicrobial OR extraction OR valorization) | March 2026 |
| Web of Science | TS = (“Lavandula angustifolia” OR lavender) AND TS = (“essential oil” OR polyphenols OR phenolics OR flavonoids OR biomass OR “post-distillation residue”) AND TS = (pharmacology OR anxiolytic OR antioxidant OR anti-inflammatory OR antimicrobial OR extraction OR valorization) | March 2026 |
| ScienceDirect | (“Lavandula angustifolia” OR lavender) AND (“essential oil” OR polyphenols OR biomass OR pharmacology OR anxiolytic OR antioxidant OR anti-inflammatory OR valorization) | March 2026 |
| SpringerLink | “Lavandula angustifolia” OR lavender AND “essential oil” OR polyphenols OR biomass OR pharmacology OR anxiolytic OR antioxidant OR anti-inflammatory OR valorization | March 2026 |
| Google Scholar | Iterative combinations of search terms including: “Lavandula angustifolia”, lavender, essential oil, polyphenols, biomass, pharmacology, anxiolytic, antioxidant, anti-inflammatory, and valorization | March 2026 |

Note: Search syntax was adapted to the indexing structure and query functionality of each database platform while maintaining conceptual consistency across search domains.


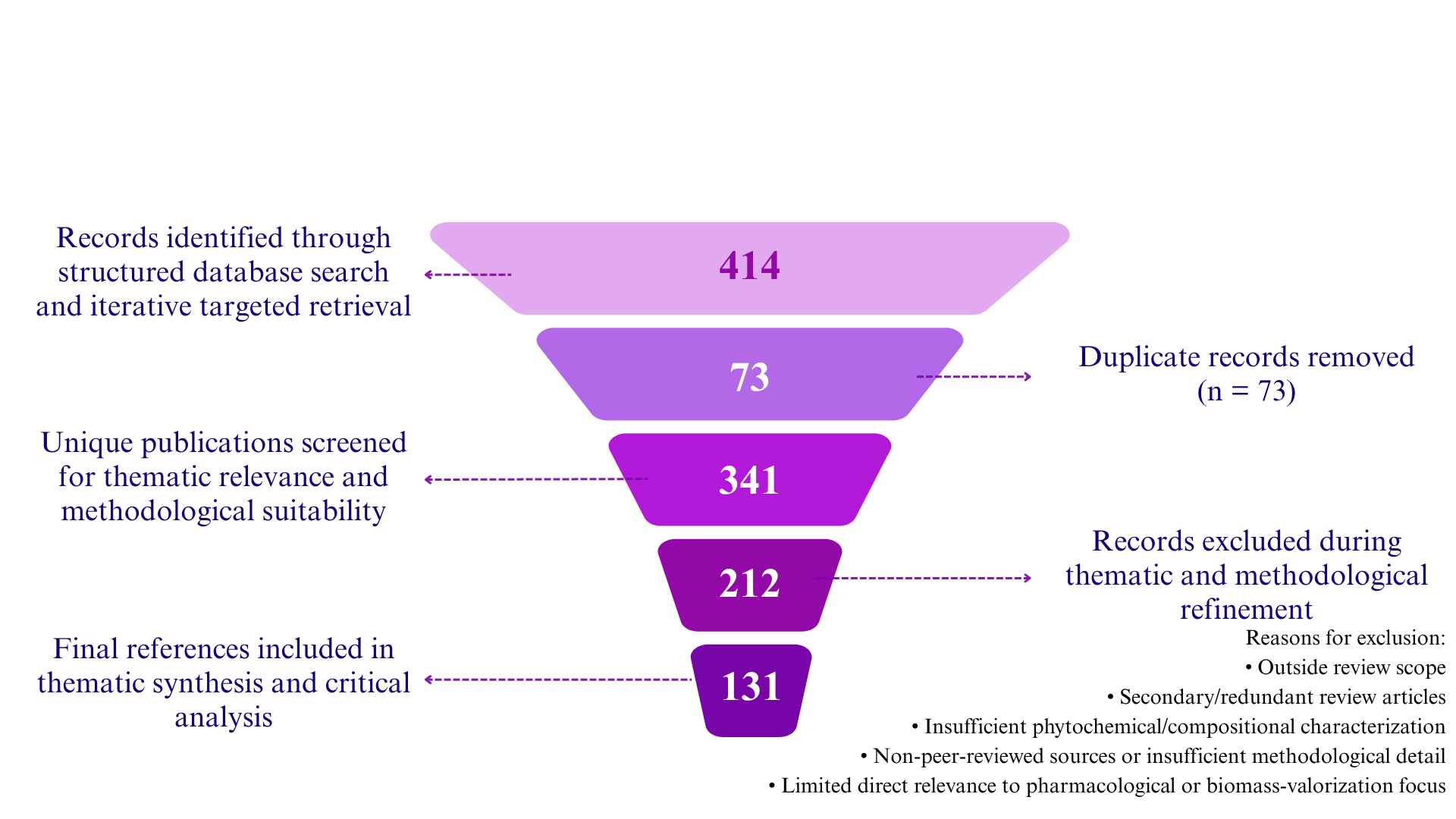


Supplementary Figure S1. Literature identification, screening, and refinement workflow applied in the present structured narrative review of *L. angustifolia*.

Note: One additional methodological reference related to narrative review methodology was included in the final bibliography but was not counted as part of the thematic evidence synthesis.
